# The wettability of complete denture base materials constructed by conventional versus digital techniques: an in-vitro study

**DOI:** 10.1186/s12903-024-04800-x

**Published:** 2024-09-13

**Authors:** Kenda I. Hanno, Nayrouz Adel Metwally

**Affiliations:** Department of Prosthodontics, Faculty of Dentistry, University of Alexandria, Champolion St, Alexandria, Egypt

**Keywords:** Wettability, Contact angle, Heat-polymerized, 3D-printed, CAD-CAM

## Abstract

**Background:**

Decreased salivary flow can make the patients uncomfortable with their complete dentures and affects the retention of the dentures. Milling and 3D printing have become an alternative to conventional denture construction techniques. The goal of this study was to evaluate the effect of conventional and digital techniques of the complete denture construction on the denture surface wettability with distilled water and saliva substitute before and after thermocycling.

**Methods:**

A total of 30 specimens were utilized in the present study. Specimens were divided according to the construction techniques into 3 groups (*n* = 10 each). Group I: Heat-polymerized polymethylmethacrylate (PMMA) group, group II: Milled group, and group III: 3-dimensional (3D)-printed group. All the specimens were subjected to 2000 cycles of thermal aging in a thermocycler. The wettability of all specimens to water and saliva substitute was measured via a contact angle goniometer (Olympus TGHM, Rame-hart Inc, USA) before and after thermocycling. Descriptive statistical analysis, plots, and the Shapiro-Wilk test were used to verify normality for each variable. One-way ANOVA was used to compare the 3 study groups, while paired samples t-test was used to compare the differences within each group (*P* < .05).

**Results:**

The smallest contact angle of drop of water to the denture base specimens before and after thermocycling were recorded in the milled group (53.0 ± 4.77 and 50.27 ± 2.30, respectively), followed by the heat polymerized PMMA group (85.65 ± 4.71 and 65.06 ± 2.27, respectively), and the 3D-printed group (91.34 ± 6.74 and 90.86 ± 8.57, respectively). While the smallest contact angle of drop of saliva substitute to denture base specimens was recorded in the milled group (56.82 ± 2.29 and 34.85 ± 7.51, respectively), followed by the 3D-printed group (72.87 ± 4.83 and 58.14 ± 9.58, respectively) and the heat polymerized PMMA group (83.62 ± 4.12 and 67.82 ± 4.93, respectively). There was statistically significant difference between the groups (*P* < .05). A significant decline in the average contact angle of drop of saliva has been reported in all groups after thermocycling. The contact angle values differed significantly between saliva substitute and distilled water in both 3D-printed and milled groups after thermocycling (*P* < .001).

**Conclusions:**

The milled denture base material presented the best wettability to water and saliva substitute than the 3D-printed and the heat-polymerized PMMA materials. Saliva substitutes improve the wetting ability of denture base materials manufactured by CAD/CAM compared with water.

## Background

Denture retention affects masticatory and speaking abilities, which affects quality of life [1]. Saliva is essential to create surface tension, cohesion, and adhesion which will eventually lead to increase in denture retention [2]. The more hydrophilic the surface of the denture is, the more easily the saliva flows over the surface and consequently better adhesive retention can be obtained [3]. The adherence of Candida albicans and other types of bacterial cells will decrease on a hydrophilic surface [4].

Saliva must flow easily and wet both the denture-bearing mucosa and the intaglio surface of the denture for proper adhesion to occur between the denture and the supporting tissues [3]. The contact angle between the liquid and the surface under test can be used to quantify wettability. Wettability has been found to have an inverse relationship with contact angle [5]. A smaller contact angle shows higher hydrophilicity, and consequently, higher wettability [6].

Wettability characteristic of the denture base material depends on the viscosity of the saliva, the purity of the adherent surface, and the shape of the irregularities on the adherent surface. The quantitative measure of the wetting process is taken to be the contact angle that the saliva makes with the adherent surface. The smaller the contact angle, the greater the wettability [7].

Conventional denture base resins have been fabricated using heat-polymerized polymethyl methacrylate (PMMA), which was the material of choice for denture construction [8]. PMMA is commonly used material due to its ease of application and accessibility. However, it has been reported that some patients showed intolerance to monomers present in acrylic materials [9]. PMMA contains carboxylate and methyl ester groups that increases its hydrophilicity and free surface energy [8]. However, additives, such as accelerators, initiators, crosslinking agents, colorants and fillers may affect its physical and chemical properties [10]. Heat polymerizing of PMMA resin causes monomer vaporizing at high processing temperatures that affects the surface and causes irregularities [11].

Complete dentures can now be constructed digitally as an alternative to traditional methods due to the advent of computer-aided design and computer-aided manufacturing (CAD/CAM) in dentistry [12]. The CAD/CAM denture bases are milled from previously polymerized highly condensed resin blanks [13]. Therefore, they are likely less porous with smoother denture surfaces than the conventional ones [14, 15].

3D printing allows construction of multiple dentures simultaneously thereby, saving time and energy [16]. Moreover, less material is wasted in comparison with milling [17]. However, high surface roughness have been reported when compared to the milled dentures [18].

Sri H et al. [19] have compared the wettability of CAD/CAM versus conventional denture bases and concluded that 3D-printed dentures had the highest contact angle, and that the wettability was lower in artificial saliva substitute than in distilled water. Another study showed no difference in wettability with distilled water and artificial saliva substitue [20].

Complete dentures are exposed to different temperature changes and loads during functional service intraorally, that may affect their mechanical and physical properties [21]. Paradowska-Stolarz et al. [22] studied the effect of aging on the mechanical properties of 3D-printed resin and they concluded that artificial aging has significantly affected both the compressive and the tensile strength of the material. It was recommended to polish the material so that its durability will be increased [22]. Research has shown that as a denture is used for a longer time, the wettability improves, therefore retention increases [23]. Studies have investigated how thermocycling affects the mechanical characteristics of denture base materials [10, 24, 25]. Tasin et al. [25] evaluated conventional, CAD/CAM milled, and 3D-printed interim materials and they found better results with digitally fabricated denture base materials than conventionally polymerized materials. In the contrary, Atalay et al. [10] have also evaluated the effect of thermocycling on the water contact angle to different materials used for denture bases and they concluded that, surface treatment significantly affected the contact angle following thermocycling, thus producing more hydrophobic surfaces with better results reported in the milled group. On the other hand, Jadhav et al. [24] found that contact angle values were not affected by thermocycling. Several studies have explored the wettability of conventional denture base materials [3, 10, 11, 19, 20], yet there is a lack of sufficient data on the wettability of digital denture base resins.

The present study has been conducted to evaluate the effect of the construction technique on the wettability of denture base materials by using both distilled water and saliva substitute prior to and following thermocycling. The first null hypothesis was that the construction technique would not influence the wettability of the denture base material by distilled water and saliva substitute. The second hypothesis was that the thermocycling would not influence the wettability of the denture base material fabricated by digital and conventional techniques.

## Methods

In this study, the wettability of distilled water and artificial saliva substitute to denture base resins constructed by heat-polymerization, CAD/CAM milling, and 3D printing, was tested.

### Sample size calculation

The specimen sample size (*n* = 60) was estimated assuming a study power of 80% and an alpha error of 5%.^19^ Eight samples were determined to be the minimum required sample size, which was raised to 10 samples to account for errors in laboratory processing. The total required sample size = number of subgroups × number per subgroup × number of groups = 3 × 10 × 2 = 60 samples [10, 26].

The software (Autodesk Meshmixer; Autodesk Inc, USA) was used to design disk-shaped specimens (10 × 2 mm) [10]. The design was then saved as standard tessellation language (STL) files.

### Specimen preparation

For the heat-polymerized group, the specimens were milled in wax (Wax white 95H10; Zirkonzahn, Germany), which were then flasked, boiled out, packed with heat-polymerized PMMA (Meliodent; Kulzer, Germany) and cured at 74ºC for 1.5 h and then at 100ºC for one hour [27]. The specimens were then deflasked and flashes were removed. A digital vernier caliper (Kawasaki, Mitutoyo Co., Japan) was used to measure the dimensions of the specimens.

For the milled group, a computer numeric controlled (CNC) milling machine (Ceramill motion 2; Amann Girrbach, Germany) was used to mill the specimens from pre-polymerized resin blocks (M-PM, Merz dental, Germany). For the construction of the 3D-printed group, the specimens were printed using a 3D printer (Form 3; Formlabs, USA) with biocompatible resin for denture bases (Denture base LP; Formlabs, USA). The software program of the printer (PreForm; Formlabs, USA) was used to add supports to the specimens, and the specimens were positioned at an angle of 45 degrees to the printer platform [28]. The thickness of each layer was adjusted to 50 μm. Following printing, the washing of the specimens was done in 99% isopropyl alcohol for 10 min, then subjected to post polymerization for 30 min using ultraviolet light in a lightbox (Form Cure; Formlabs, USA) [28].

The specimens were cleaned using a household soap and then alcohol (Ethanol, Sigma-Aldrich, Merck KGaA, Germany) to eliminate any soap residue before being submerged in an ultrasonic cleaner (Sonorex RK; Bandelin electronic GmbH, Germany) for 15 min to eliminate any contaminants from the test surface [11]. Following cleaning, the specimens were dried for 30 min at 44 °C in an oven before being cooled to room temperature [29, 30]. Each specimen was assigned a sequential number.

### Wettability measurement

The wettability was assessed by measuring the contact angles of the distilled water to the surface of each specimen by using the sessile drop method via a standard type of contact angle goniometer (Olympus TGHM, Rame-hart Instrument Co., USA). The acrylic resin specimen was positioned in the middle of the table, directly beneath the needle of a clean syringe fitted into a metal housing. To standardize the liquid used for each drop, the metal housing featured a knob on its superior aspect and a microliter graduation. Liquid was released through the needle by turning the knob in a clockwise direction [30]. The software program (Windrop ++, GBX Scientific LTD, Ireland) on the computer allowed the drops on the specimens to be seen on the screen.

**Fig. 1:**
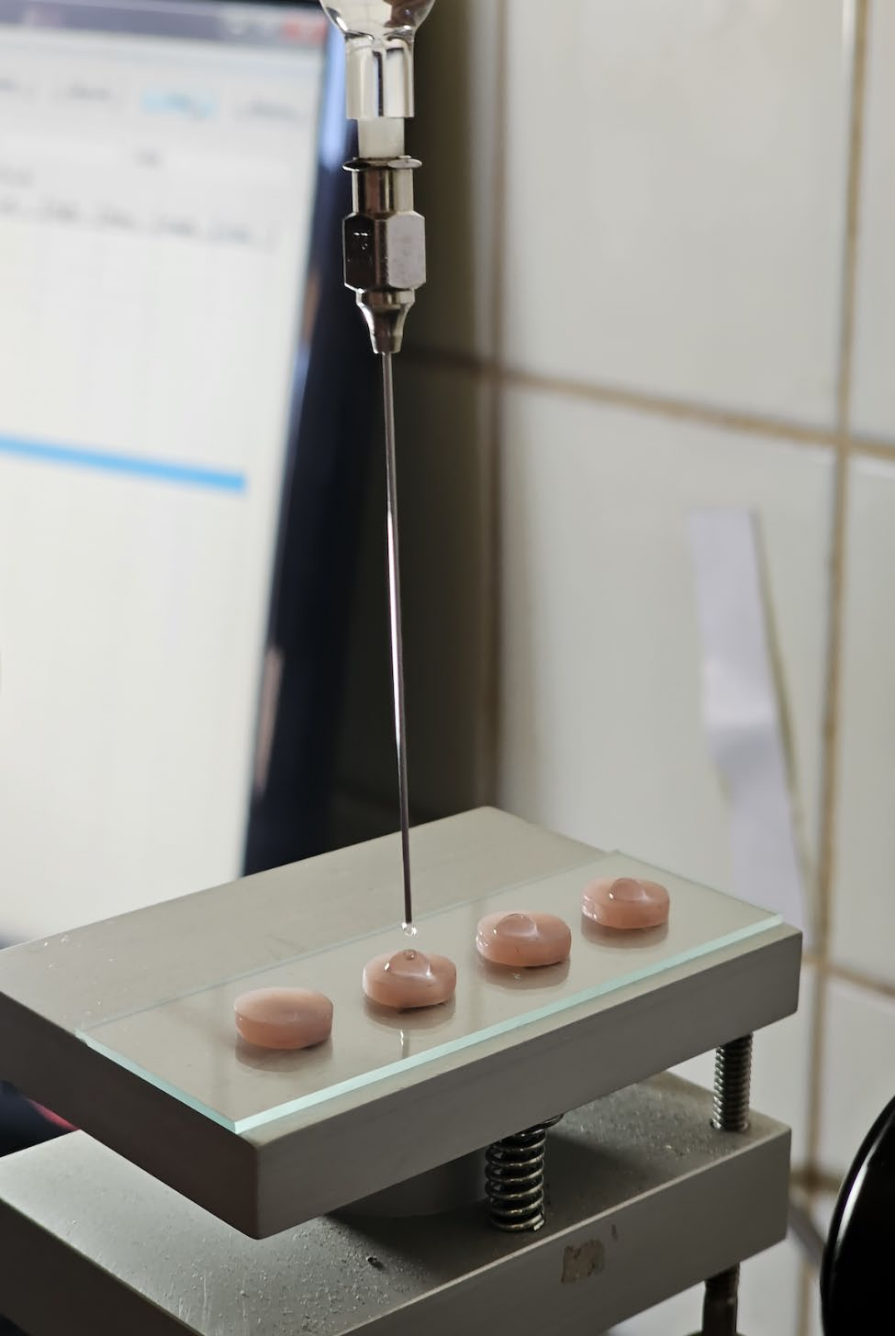
Droplets applied to the surface of each specimen on the contact angle goniometer

Deionized water droplets (4 µl) were applied on the surface of each specimen (Fig. 1). The left and right static contact angles between the tangent of the water drop and the line of the specimen were instantly measured and the mean was calculated [28]. Each specimen underwent 3 successive measurements [27]. The specimens were blotted to dry between the measurements [29]. The same steps were repeated using artificial saliva substitute (Aqwet; Cipla, India).

After that, all specimens underwent 2000 cycles of thermal aging in a thermocycler (THE-1100; SD Mechatronik, Germany) to replicate the use of denture base materials in clinical settings over a period of 2 years [9]. Immersion times were 30 s in water that was 5 °C/55°C, and dwell times were 10 s [31, 32]. Following thermocycling, the specimens were dried in the same way as previously mentioned. The wettability test was performed before and after thermocycling for the 3 groups.

### Statistical analysis

Data were analyzed using IBM SPSS for Windows, Version 26.0 (IBM Corp., USA). Normality was checked for all variables by using descriptive statistics, plots, and the Shapiro–Wilk test. All variables showed normal distribution, so means and standard deviation (SD) were calculated, and parametric tests were used. Comparisons within each group were done using paired samples t-test, while comparisons between the 3 study groups were done using One-Way ANOVA followed by multiple pairwise comparisons using Bonferroni adjusted significance level. A mixed model ANOVA was performed to assess the association between group (conventional, milled, and 3D-printed resin), medium (water and saliva), and thermocycling (before and after) with contact angle. Adjusted means, standard error (SE) and 95% Confidence intervals (CI)s were calculated. Significance was set at *P* < .05.

## Results

**Fig. 2:**
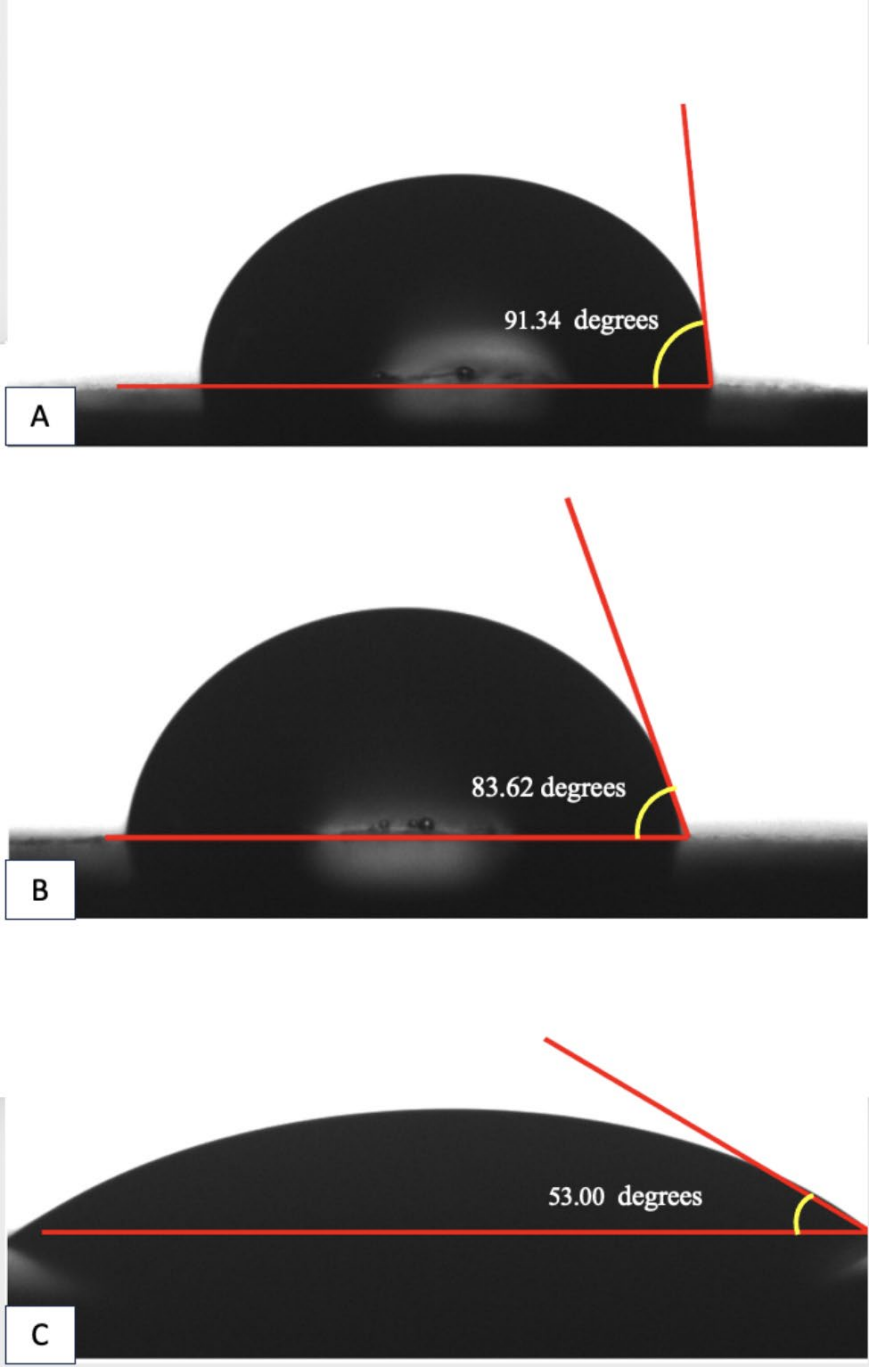
Digital images of goniometer measurements to drop of water. **A**, 3D-printed group. **B**, Heat-polymerized group. **C**, Milled group

The contact angle values of the drop of water to the denture base surface, recorded in all groups before and after thermocycling, are presented in Table 1. The contact angle values were the smallest in the milled group both before thermocycling (53.0 ± 4.77) and after thermocycling (50.27 ± 2.30), followed by the heat polymerized PMMA group (85.65 ± 4.71 and 65.06 ± 2.27, respectively), and the 3D-printed group (91.34 ± 6.74 and 90.86 ± 8.57, respectively). The differences were statistically significant (*P <* .001). Comparisons within each group were done using paired samples t-test. The contact angle values showed decrease after thermocycling in all groups with a significant effect only in the heat-polymerized group (*P <* .001) (Fig. 2).

**Table 1:** Comparison of the contact angles (degrees) of the drop of water to the denture base surface in all groups before and after thermocycling

Contact angle (degrees)	Group I Heat-polymerized resin	Group II Milled resin	Group III 3D- printed resin	*P* value 1
	Mean ± standard deviation			
Before Thermocycling	85.65 ± 4.71	53.00 ± 4.77	91.34 ± 6.74	< 0.001*
After Thermocycling	65.06 ± 2.27	50.27 ± 2.30	90.86 ± 8.57	< 0.001*
*P* value 2	*<* 0.001*	0.12	0.85	

*P* value 1: One-way ANOVA was used, *P* value 2: Paired t-test was used

*statistically significant at *P* < .05

**Fig. 3:**
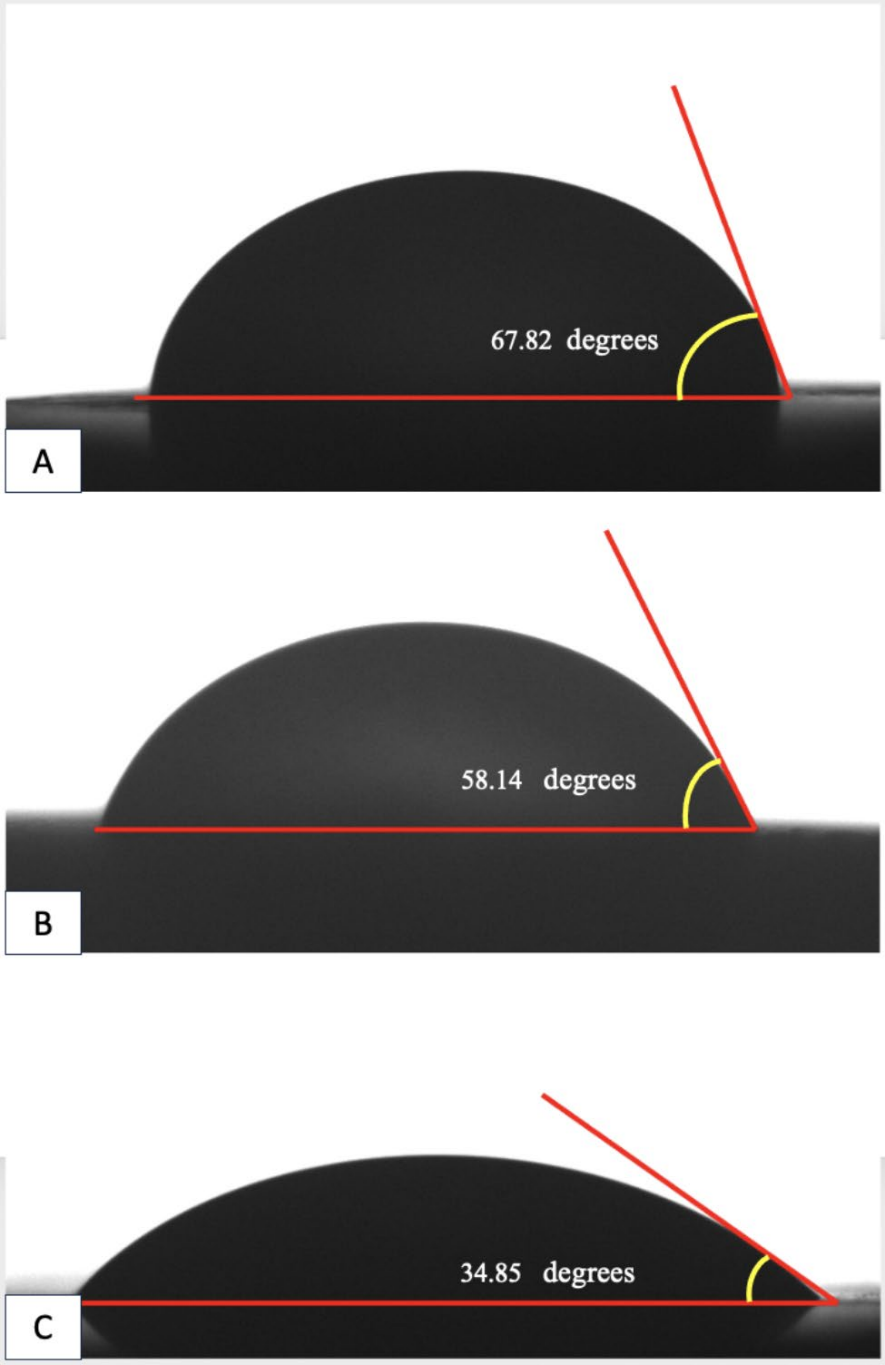
Digital images of goniometer measurements to drop of saliva substitute. **A**, Heat-polymerized group. **B**, 3D-printed group. **C**, Milled group

The contact angle values of the drop of saliva substitute to the denture base surface recorded in all groups before and after thermocycling are presented in Table 2. The smallest contact angle values were recorded in the milled group before (56.82 ± 2.29) and after thermocycling (34.85 ± 7.51), followed by the 3D-printed group (72.87 ± 4.83 and 58.14 ± 9.58, respectively), and then the heat-polymerized group (83.62 ± 4.12 and 67.82 ± 4.93, respectively). The comparison between the groups was statistically significant before and after thermocycling (*P* < .001). A significant decline in the average contact angle of the drop of saliva to the denture base surface has been reported in all groups after thermocycling (*P* < .001, *P* < .001and *P* = .004) (Fig. 3).

**Table 2:** Comparison of the contact angles (degrees) of the drop of saliva substitute to the denture base material surface in all groups before and after thermocycling

Contact angle (degree)	Group I Heat-polymerized resin	Group II Milled resin	Group III 3D-printed resin	*P* value 1
	Mean ± standard deviation			
Before Thermocycling	83.62 ± 4.12	56.82 ± 2.29	72.87 ± 4.83	< 0.001*
After Thermocycling	67.82 ± 4.93	34.85 ± 7.51	58.14 ± 9.58	0.001*
*P* value 2	< 0.001*	< 0.001*	0.004*	

*P* value 1: One-way ANOVA was used, p value 2: Paired t-test was used

*statistically significant at *P* < .05

A comparison of the contact angle produced by a drop of saliva and water to the denture base material surface in all groups before and after thermocycling is presented in Table 3. In the conventional heat-polymerized group, there was no significant differences between the contact angles with water and saliva (*P* = .46). In the milled group, the contact angles with water and saliva before thermocycling were similar (*P* = .07), however, the contact angle with saliva was significantly lower than water after thermocycling *(P < .*001). In the 3D-printed group, the contact angles with saliva were significantly lower than water both prior to and following thermocycling *(P* < .001).

**Table 3:** Comparison of the contact angle produced by drop of saliva and water to the denture base material surface in all groups before and after thermocycling

Contact angle	Group I Heat-polymerized resin		Group II Milled resin		Group III 3D-printed resin		*P* value 1
	Water	Saliva	Water	Saliva	Water	Saliva	
	Mean ± SD						
Before thermocycling	85.65 ± 4.71	83.62 ± 4.12	53.00 ± 4.77 b	56.82 ± 2.29 b	91.34 ± 6.74 c	72.87 ± 4.83 b	< .001*
*P* value 2	.46		.07		< .001*		
After thermocycling	65.06 ± 2.27	67.82 ± 4.93	50.27 ± 2.30	34.85 ± 7.51	90.86 ± 8.57	58.14 ± 9.58	< .001*
*P* value 2	.13		< .001*		< .001*		

*P* value 1: One-way ANOVA was used, p value 2: Paired t-test was used

*statistically significant at *P* < .05

Mixed-model analysis for the influence of material, medium and thermocycling on contact angle is shown in Table 4. The type of material, the media (water and artificial saliva substitute), and thermocycling had a significant effect on the contact angle values.

**Table 4:** Mixed-model analysis for the influence of material, medium and thermocycling on contact angle

		Adjusted mean (SE)	95% CI	*P* value
Material	Heat-polymerized resin	75.54 (0.91) a	73.74, 77.34	< 0.001*
	Milled resin	48.74 (0.91) b	46.93, 50.54	
	3D-printed resin	78.30 (0.91) a	76.50, 80.10	
Medium	Water	72.70 (0.74)	71.23, 74.17	< 0.001*
	Saliva	62.35 (0.74)	60.88, 63.82	
Thermocycling	Before	69.77 (1.40)	66.99, 72.54	0.03*
	After	65.28 (1.40)	62.51, 68.06	

SE: Standard Error, CI: Confidence Interval

Model F: 53.37, p value < 0.001*, Model Adjusted R2: 0.64

*P* value of interaction < 0.001*

*statistically significant at *P* < .05

a-d: different letters denote statistically significant differences between groups using Bonferroni adjusted significance level

## Discussion

The first null hypothesis was rejected as the construction technique had showed a significant effect on the wettability of the denture base material by distilled water and saliva substitute both prior to and following thermocycling. The second null hypothesis was also rejected as a significant increase in the denture wettability after thermocycling has been reported in all groups.

The wettability is an important aspect to address in the selection of the denture base material [1]. The retentive adhesive forces between the tissue surface and the denture base can only be achieved by wetting the two surfaces [3]. In the present study, thermal cycling was done to simulate the oral environment where the denture bases are usually exposed to changes in the temperature which affect their properties [23].

The best wettability results have been reported in the milled group being the best hydrophilic surface among all studied groups, possibly because of the processing protocol, where the denture bases are milled from highly condensed blanks with minimal porosity [13]. Moreover, the milling process produces a smoother surface than the conventional and 3D printing techniques [13]. The results of the current study were consistent with Steinmassl et al. [15] and Sri H et al. [19] who found that CAD/CAM techniques had affected the surface properties resulting in smoother surfaces than conventional dentures and consequently better wettability.

The results are also in line with Al-Dwairi et al. [29] who compared milled PMMA to conventional heat-polymerized resins and found better wettability in the milled group. El-Samahy et al. [28] investigated the surface roughness of both CAD/CAM milled and 3D-printed specimens from the same manufacturers as those used in the present study, and they concluded that milled specimens had lower surface roughness. This explains why wettability is higher in the milled group in the present study.

Water cannot moisten and lubricate the oral mucosa adequately. However, it can be used as a lubricant if needed [5]. Speaking, swallowing, and mastication are all difficult for patients with hyposalivation. Dry mouth increases the susceptibility of the mucosa to irritation and epithelial atrophy, which can result in ulceration, fissuring, and inflammation [5].

Artificial saliva substitutes have been used for these patients to increase lubrication of the oral cavity and improve retention of complete dentures. The results of the current study showed improved wettability results with the saliva substitute in comparison to the water droplets. This may be attributed to the type of the artificial saliva substitute used which was carboxymethylcellulose (CMC) based and has been concluded by Jaiswal et al. [30] to have the best wettability results than other saliva substitute types used in their study and was similar to the human saliva.

The contact angles of the conventional heat-polymerized group to both water and saliva were similar in the present study. These results are consistent with Ramanna et al. [20] who revealed that the contact angles between water and a CMC-based saliva substitute were not significantly different.

However, with the milled and 3D-printed group, the contact angles with artificial saliva substitute were significantly lower than distilled water, indicating better wettability with artificial saliva substitute. These results are consistent with Jadhav et al. [24], who compared the wettability with saliva substitute and distilled water and revealed improved wettability with artificial saliva susbtitute in heat polymerized, microwave polymerized resin, and injection molded polyamide. These results are also similar to Lai Q et al. [5], who found lower contact angles with artificial and human saliva compared with distilled water. However, the results are opposite to Sri et al. [19], who revealed higher wettability with distilled water than with artificial saliva substitute. This could be due to different testing conditions.

Accelerated artificial aging by thermal cycling is a method that involves immersion in water and in temperature under standard conditions performed in the laboratory [21]. The results in the current study showed that the contact angles with the water droplets were reduced in all groups after thermocycling, with a significant difference only in the heat-polymerized group.

The contact angle with the artificial saliva substitute was also significantly reduced after thermocycling in all the 3 groups indicating improved retention of the dentures. Those results were consistent with Tasin et al. [25], who had found better retention results in both conventional and CAD/CAM denture bases after thermocycling, as wearing the denture for a longer time is supposed to increase its retention [1]. However, these results were opposite to Jadhav et al. [24], who found that thermocycling did not significantly affect the contact angle values.

Milling technique can be promising in construction of denture base materials, especially in patients who are suffering from reduced salivary flow due to its high surface wettability when compared to 3D printing and conventional construction techniques.

Highly promising chitosan coating material has been suggested to be used in the dental field due to its bactericidal effect [33]. Further studies are recommended to evaluate the effect of this coating on the wettability of the denture surface.

After conducting this study, some limitations were drawn, as the in vitro design that may not have simulated the oral environment. The specimens tested were flat and will not duplicate the non-uniform surfaces of the dentures which might influence the wettability. Further clinical studies are required to study the effect of oral conditions and the intaglio surfaces of the dentures on wettability.

## Conclusions

Based on the findings of this in vitro study, the following were concluded:

1. The technique of fabricating the denture base material has a significant effect on the wettability of its surface.
2. The milled denture base material showed a significantly better wettability to the water and the saliva substitute than the 3D-printed and the heat polymerized PMMA denture base materials.
3. Thermocycling has significantly improved the wettability to saliva substitute in all groups.
4. Saliva substitute allowed higher wettability to be achieved in CAD/CAM denture base materials compared with water. Saliva substitutes improve the wettability of denture base resins and hence, enhance denture retention.

## Data Availability

The raw data of the present study is available at: https://figshare.com/articles/dataset/_b_Evaluation_of_the_wettability_of_complete_denture_base_materials_constructed_by_conventional_and_digital_techniques_An_in-vitro_study_b_/24763686.

## Abbreviations

3D: Three-dimensional
CAD-CAM: computer-aided design and computer-aided manufacturing
PMMA: Polymethylmethacrylate
CNC: Computer numerically controlled
